# Notes on Preparations for the Sick

**Published:** 1888-12

**Authors:** 


### Notes on Preparations for the Sick.

**Oleum Iodi.**—THE BLYTHESWOOD CHEMICAL COMPANY, Govan.

Messrs. Lorimer & Co. send us a sample of this, which is a solution of pure iodine, in about equal parts of vegetable oil and water. It contains ten grains of iodine in each fluid ounce, is intended for external use as an embrocation in the incipient stage of phthisis, chronic bronchitis, and other chest affections; also in chronic rheumatism, and in cases of scrofulous gland-swellings. The solution appears to have several advantages over the ordinary iodine paints: it does not stain the skin; it does not irritate, blister, or cause desquamation; and it must be more penetrating in its action. We should expect it to be especially useful in cases of ringworm of the scalp, and in other conditions where a penetrating germicide is desirable.

**Pumilio Pine Products.**—G. & G. STERN, London.

These are prepared from the species of pine which grows at altitudes of six to eight thousand feet above the sea, above the line of perpetual snow, where the stunted trees contain much more of the pumiline essence than those growing in warmer and less elevated regions. As it is scarcely possible that the patients should live amongst the growing pines, the products of the latter are transported to the patient elsewhere, and may be obtained in the form of **Pumiline Essence**, for inhalation; **Pumiline Extract**, for baths; **Jujubes**, &c. Their efficacy is now being fully recognised, inasmuch as the rapidly increasing demand for them is the best proof of their value.

**Gunn's Oat Flour.**—GEORGE GUNN, Bath.

This food is a useful addition to the dietary of the sick. It is quite different from the ordinary product of the oat, and is liked even by those who do not like oatmeal as commonly sold and prepared.

**Cod Liver Oil Emulsion, with Hypophosphites.**—T. BUXTON, Bristol.

This is a carefully prepared form of this excellent combination. Its usefulness in a variety of chronic disorders is thoroughly established.

From Mr. Buxton we have also received a sample of Ingram and Royle's **Natural Karlsbad Sprudel Salt**, which, elegantly mounted in glass bottles of 100 or 200 grammes, "gets produced in the salt administration of the Municipality of Karlsbad, in their own Sprudel Salt-works." It is too well-known and appreciated to require either comment or commendation.

---

**De Miel's Perfect Health Biscuits.**—INTERNATIONAL PATENTS ASSOCIATION, London.

These biscuits are well-made, palatable, and nourishing. In addition to the ordinary nitrogenous and carbo-hydrate elements, each biscuit contains five grains of combined phosphates.

---

**Patent Poison Bottle.**—E. S. HERMES, London.

This is an arrangement by which it is impossible for the most careless or ignorant person to administer poisonous lotions or liniments internally. The only opening is at the bottom of the bottle: accordingly, before the contents can be poured out, the bottle must be reversed. It possesses the disadvantage that after use it must each time be carefully wiped, as it has to stand upon the part from which the fluid has been poured.

Messrs. Parke, Davis & Co., of Detroit, Michigan, U.S.A., request us to intimate that Mr. W. H. Phillips is not any longer authorised to take any orders or to transact business of any kind for them.

